# Mapping landscape canopy nitrogen content from space using PRISMA data

**DOI:** 10.1016/j.isprsjprs.2021.06.017

**Published:** 2021-07-15

**Authors:** Jochem Verrelst, Juan Pablo Rivera-Caicedo, Pablo Reyes-Muñoz, Miguel Morata, Eatidal Amin, Giulia Tagliabue, Cinzia Panigada, Tobias Hank, Katja Berger

**Affiliations:** a Image Processing Laboratory (IPL), Parc Científic, Universitat de València, València, Spain; b Secretary of Research and Graduate Studies, CONACYT-UAN, Tepic, Nayarit, Mexico; c Remote Sensing of Environmental Dynamics Laboratory, University of Milano - Bicocca, Milano, Italy; d Department of Geography, Ludwig-Maximilians-Universitaet Munich, Munich, Germany

**Keywords:** Canopy nitrogen content, PRISMA, CHIME, Hybrid retrieval, Gaussian process regression, Dimensionality reduction, Active learning, Imaging spectroscopy

## Abstract

Satellite imaging spectroscopy for terrestrial applications is reaching maturity with recently launched and upcoming science-driven missions, e.g. PRecursore IperSpettrale della Missione Applicativa (PRISMA) and Environmental Mapping and Analysis Program (EnMAP), respectively. Moreover, the high-priority mission candidate Copernicus Hyperspectral Imaging Mission for the Environment (CHIME) is expected to globally provide routine hyperspectral observations to support new and enhanced services for, among others, sustainable agricultural and biodiversity management. Thanks to the provision of contiguous visible-to-shortwave infrared spectral data, hyperspectral missions open enhanced opportunities for the development of new-generation retrieval models of multiple vegetation traits. Among these, canopy nitrogen content (CNC) is one of the most promising variables given its importance for agricultural monitoring applications. This work presents the first hybrid CNC retrieval model for the operational delivery from spaceborne imaging spectroscopy data. To achieve this, physically-based models were combined with machine learning regression algorithms and active learning (AL). The key concepts involve: (1) coupling the radiative transfer models PROSPECT-PRO and SAIL for the generation of a wide range of vegetation states as training data, (2) using dimensionality reduction to deal with collinearity, (3) applying an AL technique in combination with Gaussian process regression (GPR) for fine-tuning the training dataset on in field collected data, and (4) adding non-vegetated spectra to enable the model to deal with spectral heterogeneity in the image. The final CNC model was successfully validated against field data achieving a low root mean square error (RMSE) of 3.4 *g/m^2^* and coefficient of determination (*R*^2^) of 0.7. The model was applied to a PRISMA image acquired over agricultural areas in the North of Munich, Germany. Mapping aboveground CNC yielded reliable estimates over the whole landscape and meaningful associated uncertainties. These promising results demonstrate the feasibility of routinely quantifying CNC from space, such as in an operational context as part of the future CHIME mission.

## 1 Introduction

With current and upcoming satellite imaging spectroscopy missions, unique data streams of hyperspectral measurements from the Earth surface will be provided in almost real-time. After the two initial experimental Hyperion/EO-1 and CHRIS/PROBA missions, two primarily science-driven spaceborne sensors, such as the launched PRecursore IperSpettrale della Missione Applicativa (PRISMA) (Loizzo et al., 2019) and planned Environmental Mapping and Analysis Program (EnMAP) (Guanter et al., 2015), started to pave the way for future scientific and operational hyperspectral missions. Among these missions are the FLuorescence EXplorer (FLEX) (Drusch et al., 2017), the NASA Surface Biology and Geology observing system (SBG) (National Academies of Sciences, 2018) and the Copernicus Hyperspectral Imaging Mission for the Environment (CHIME) (Nieke and Rast, 2019). Apart from FLEX being a scientific mission dedicated to vegetation fluorescence retrieval, all these hyperspectral missions will observe reflected sunlight across a wide range of wavelengths from visible to shortwave infrared (SWIR) domains (“VSWIR”, approximately 400–2500 nm) with the purpose of providing mapping and monitoring services for multiple civil and environmental domains. Effectively, exploitation of hyper-spectral data enables quantitative estimation of key biophysical and biochemical variables beyond relative vitality indicators that were also provided by conventional broadband satellite missions (Hank et al., 2019). Agriculture has been identified as a key domain where repetitive hyperspectral data can provide up-to-date and unique spatiotemporal information about the crop status and development (Hank et al., 2019). Thanks to imaging spectroscopy technology, the focus widened from estimating structural variables and chlorophyll pigments towards the quantification of specific plant compounds and nutrients, such as nitrogen (N) (Homolová et al., 2013). N strongly influences crop growth and quality, and hence only with the determination of crop N status, proper fertilizer management can be realized. In this respect, mapping of crop or canopy nitrogen content (CNC) from remote sensing data is considered as an efficient way to enable site-specific fertilization measures, and consequently assuring high quality grain production (Lemaire et al., 2008; Baret et al., 2007).

When it comes to N mapping, the majority of reported methods relied on the apparently strong link between chlorophyll content and N (Berger et al., 2020). However, within a plant, chlorophyll pigments contain only a small fraction of N, representing less than 2% of the total leaf N (Kokaly et al., 2009). For this reason, the chlorophyll–N relationship often showed only moderate correlations between species and growth stages across ecosystems (Homolová et al., 2013). Instead, proteins are the major nitrogen-containing biochemical constituents, with rubisco holding up to 50% of N in green leaves (Chapin et al., 1987; Elvidge, 1990; Kokaly et al., 2009). Moreover, leaf chlorophyll content decreases after mature growth stages whereas leaf N is translocated to other plant organs, such as fruits. This leads to a nonlinear relationship between leaf chlorophyll content and plant nitrogen throughout the growth cycle (Berger et al., 2020). The benefit of using protein-related spectral wavelengths in the SWIR for N retrieval was confirmed by several studies (Dunn et al., 2016; Herrmann et al., 2010), relying on the more robust link between N and proteins.

A recent review paper summarized earlier attempts and provided a pathway towards successful CNC mapping (Berger et al., 2020). In short, it was proposed that: (1) instead of the relationship between N and leaf chlorophyll content, leaf protein content is a more meaningful proxy for N (Féret et al., 2021); (2) these relationships should be primarily exploited in the SWIR (1300–2500 nm) due to absorption features of proteins located in this spectral domain (Curran, 1989), implying that the SWIR is more successful for CNC retrieval than the conventional visible to near infrared (VNIR) region, and (3) imaging spectroscopy sensors should be preferred over multispectral sensors, since they provide the required continuous spectral coverage to capture the subtle spectral signatures related to proteins, (e.g. Herrmann et al., 2010; Serrano et al., 2002). Moreover, recent progress in leaf optical properties modeling whereby spectral decomposition of leaf dry matter content into nitrogen-based proteins and other carbon-based constituents (CBC) has been explicitly parameterized, opened the path to develop physically-sound CNC retrieval models (Wang et al., 2018; Féret et al., 2021). Pursuing this research line for mapping applications, some experimental studies have already demonstrated the feasibility of producing local CNC maps from airborne hyperspectral data (Berger et al., 2020; Camino et al., 2018; Verrelst et al., 2020; Wang et al., 2018). Though we are on the verge of gaining access to routinely acquired hyperspectral images from space, the first landscape CNC map obtained from spaceborne imaging spectroscopy data based on the physical protein-N relation is still to be awaited for.

The first new-generation spaceborne sensor that meets the requirements for this application is PRISMA of the Italian Space Agency (ASI). The PRISMA spacecraft, which was launched on 22 March 2019 into its target sun-synchronous orbit, represents a pre-operational and technology demonstrator mission (Rast and Painter, 2019). The mission aims to offer data for multiple applications within environmental monitoring and resources management, among those agriculture. The design of the sensor is based on a pushbroom type concept providing hyperspectral data in 239 bands at variable spectral bandwidths between 6 and 12 nm. Ground sampling distance (GSD) is 30 m and data on a 30-km swath are provided with an orbit repeat cycle of 29 days (Loizzo et al., 2019). Following the precursor missions like PRISMA and EnMAP, the CHIME sensor will be designed with the goal to provide routine hyperspectral observations through the Copernicus Programme (Rast and Painter, 2019), hence complementing the multispectral Sentinel-2 mission starting between 2025 and 2030 (Ustin and Middleton, 2021). CHIME is foreseen to provide imaging spectroscopy data with high radiometric accuracy in the range from 400–2500 nm (over 200 bands in appr. 10 nm width), with a GSD of 30 m and repeat cycle of 22 (11) days with 1 (2) satellite(s) in a sun synchronous orbit. Moreover, a set of downstream-products, among others vegetation functional traits, will be offered to users as part of the mission catalogue to encourage the operational use of the data. To create these downstream products, new-generation retrieval methods need to be prepared for operational processing of spaceborne hyperspectral data. In this respect, hybrid workflows have evolved as one of the most promising approaches (Verrelst et al., 2019; Brede et al., 2020): these methods blend physics described by radiative transfer models (RTM) with the speed and efficiency of machine learning (ML) algorithms. Within such a scheme, training data sets are generated from RTM simulations. Then, the ML algorithm learns the (nonlinear) relationship between the pairs of reflectance and vegetation trait of interest. These training data bases have to fulfill the pre-requisites of representing the canopy structural and biochemical properties realistically on the one hand, and of being small enough to avoid long training and run-time required by some ML regression algorithms on the other. Accordingly, this approach demands for a balanced training dataset with a trade-off between optimized information content in respect to the full data set, and a minimum amount of samples.

Earlier work in preparation of the scientific FLEX fluorescence mission paved the path of hybrid models in an operational imaging spectroscopy context (De Grave et al., 2020). As part of the FLEX processing chain, a hybrid workflow was developed for the retrieval of established essential biophysical and biochemical variables, such as leaf chlorophyll content *(C_a_b*), leaf area index (LAI), fraction of absorbed photosynthetically active radiation (FAPAR) and fractional vegetation cover (FVC). The pursued approach was to generate a training data base from the RTM Soil Canopy Observation, Photochemistry and Energy fluxes (SCOPE) (Van der Tol et al., 2014), which was used to train Gaussian process regression (GPR) (Rasmussen and Williams, 2006) algorithms. GPR as a probabilistic ML was preferred as key algorithm due to its ability to provide associated uncertainty estimates along with the predictions. This special feature enables to assess the fidelity of developed models when transferring them into other space and times, thus reducing the need of reference data collections for model calibration and validation (Verrelst et al., 2013). Just as any other regression algorithm, GPR suffers from spectral collinearity when too many similar bands are fed into the algorithm. To circumvent collinearity, an elegant solution is to apply dimensionality reduction (DR) techniques, typically principal component analysis (PCA) (Rivera-Caicedo et al., 2017; Jolliffe and Cadima, 2016). Hence, combining simulated training data together with PCA and GPR allows the development of hybrid models that enable fast processing of hyperspectral data into vegetation traits. This concept of training hybrid models with principal components instead of original spectra was previously successfully tested and applied in earlier model developments using GPR (De Grave et al., 2020) and other ML algorithms (e.g. neural networks) (Danner et al., 2021) for hyperspectral data conversion into vegetation products.

Apart from DR in the spectral domain, further gain in mapping performance and processing speed can be obtained by exploring DR in the sampling domain. A solution to the sampling reduction problem is given by semi-supervised approaches, in which unlabeled samples are exploited during the design of the regression model (Pasolli et al., 2012). These techniques are also known as active learning (AL), aiming to optimize training datasets through intelligent sampling by means of an iterative procedure. The progress of AL methods for terrestrial vegetation traits estimations from Earth observation data has been summarized in a review paper (Berger et al., 2021).

Altogether, with the ambition of contributing to the planned CHIME mission by developing efficient retrieval models, a similar workflow is proposed, yet customized with hybrid models using AL for the retrieval of a variety of vegetation traits (Verrelst et al., 2021). In this context, a range of established vegetation traits will be provided (De Grave et al., 2020), but also more challenging traits which can only be obtained from hyperspectral data, such as CNC will be targeted (E.S.A., 2019). CNC is probably the most important variable to quantify in an agricultural context; nonetheless, to the best of our knowledge, CNC has not yet been routinely retrieved from space due to missing operational satellite imaging spectroscopy missions in the past. In this respect, the PRISMA mission provides an ideal benchmark for the demonstration of these new-generation retrieval algorithms. Specifically, this brings us to the following objectives of our study: (1) to develop a hybrid retrieval strategy targeting CNC based on a list of defined criteria; and (2) to test mapping capabilities of the final CNC retrieval model by applying it to a PRISMA scene. With this work we aim to present the first landscape CNC map from space with an algorithm generally applicable in the operational context of the future CHIME mission.

## 2 Material & methods

### 2.1 Experimental site and imaging spectroscopy data

#### 2.1.1 Munich-North-Isar campaigns

**Fig. 1:**
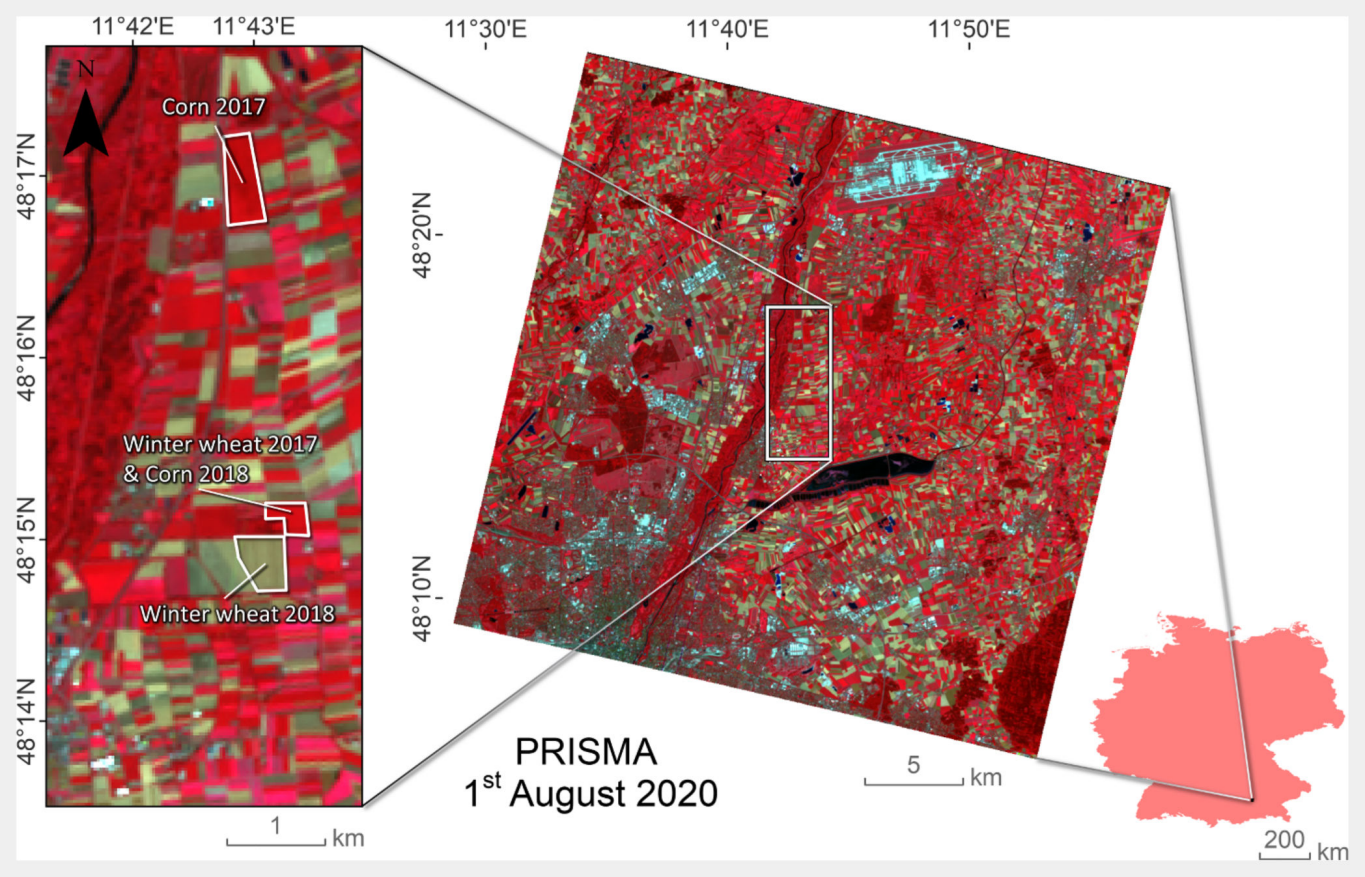
PRISMA illustration of the North of Munich and zoom-into MNI test site with winter wheat and corn fields (2017 and 2018), shown in false color-infrared (R: 865.6 nm, G: 650.5 nm, B: 554.3 nm). Provided by Matthias Wocher, LMU Munich.

For our study, data of the German Munich-North-Isar (MNI) campaigns located in the North of Munich, in Southern Germany (N 48° 16’, E 11°42’) were explored. MNI represents an agricultural consolidated long-term test site for the preparation of the future EnMAP mission (Danner et al., 2019; Wocher et al., 2018). EnMAP will cover a 30-km-wide area in the across-track direction with GSD of 30 m and 242 bands ranging from 400 to 2500 nm, which is very alike to PRISMA and future CHIME missions. With the relatively high signal-to-noise ratio around 180:1 and a spectral sampling interval around 10 nm in the SWIR, the sensor also provides optimal features for CNC retrieval based on the N - protein relationship (Berger et al., 2020). At the MNI site, extensive weekly trials including field spectroscopy and destructive measurements were carried out on winter wheat *(Triticum aestivum*) and corn (*Zea mays*) fields during the growing periods of 2017 and 2018 (see Fig. 1). In each field, a 30 × 30 m grid of nine 10 x 10 m squares was marked out corresponding to the elementary sampling unit (ESU) of a future EnMAP pixel, which also coincides with the spatial resolution of PRISMA data. Hyperspectral signatures of the canopy (within the 350–2500 nm range) were measured before the biomass sampling at each date using the Analytical Spectral Devices Inc. (ASD; Boulder, CO, USA) FieldSpec3 JR Spectroradiometer. Specifically, sampling consisted of five nadir measurements per ESU at a stable height above the canopy using a 25° field of view of the fiber optic cable. During the measurements, the sensor was slowly moved over the crop target while keeping the nadir angle. This procedure was required to obtain representative spectral signals capturing the full heterogeneity of the canopy including soil background reflectance. Finally, all spectra from the ESUs were averaged to obtain a representative value for the pseudo EnMAP pixel. Processing of proximal spectral sensing data included removal of bands in the water absorption region, splice-correction, white reference baseline calibration, and slight smoothing using a Savitzky-Golay filter (Wocher et al., 2018). Collected spectral data were subsequently resampled to the PRISMA band settings using full width at half maximum (FWHM) Gaussian information.

For N determination, wheat plants covering an area of 0.25 m^2^ and three corn plants were cut and weighed. Row distance and plants per meter were recorded simultaneously. Biomass samples were brought to the lab, where the combustion method was applied using the elemental analyzer vario EL cube (Elementar, Germany). For this, samples were oven-dried at 105°C until constant dry weight could be determined after 24 h (Berger et al., 2020). Samples were grinded and N concentration (N %), being mass of absorbing materials (dry matter) per unit dry mass in [mg/g] or [%], was measured. Aboveground N content in [g/m^2^] was finally calculated by multiplying N% with plant organ-specific dry mass per unit ground area in [g/m^2^]. A total number of 30 measurements was available for validation, composed of leaves plus stalks N content of wheat and corn. Table 1 indicates the crop type, dates of biomass sampling, growth stages, measured ranges, mean values and standard deviations (SD) of aboveground N content for leaves plus stalks.

Due to the inability of radiation to detect N content of thick tissues, measured CNC of fruits, present at mature growth stages, was excluded from our validation data set (Berger et al., 2020) (see also discussion section).

#### 2.1.2 PRISMA data

**Fig. 2:**
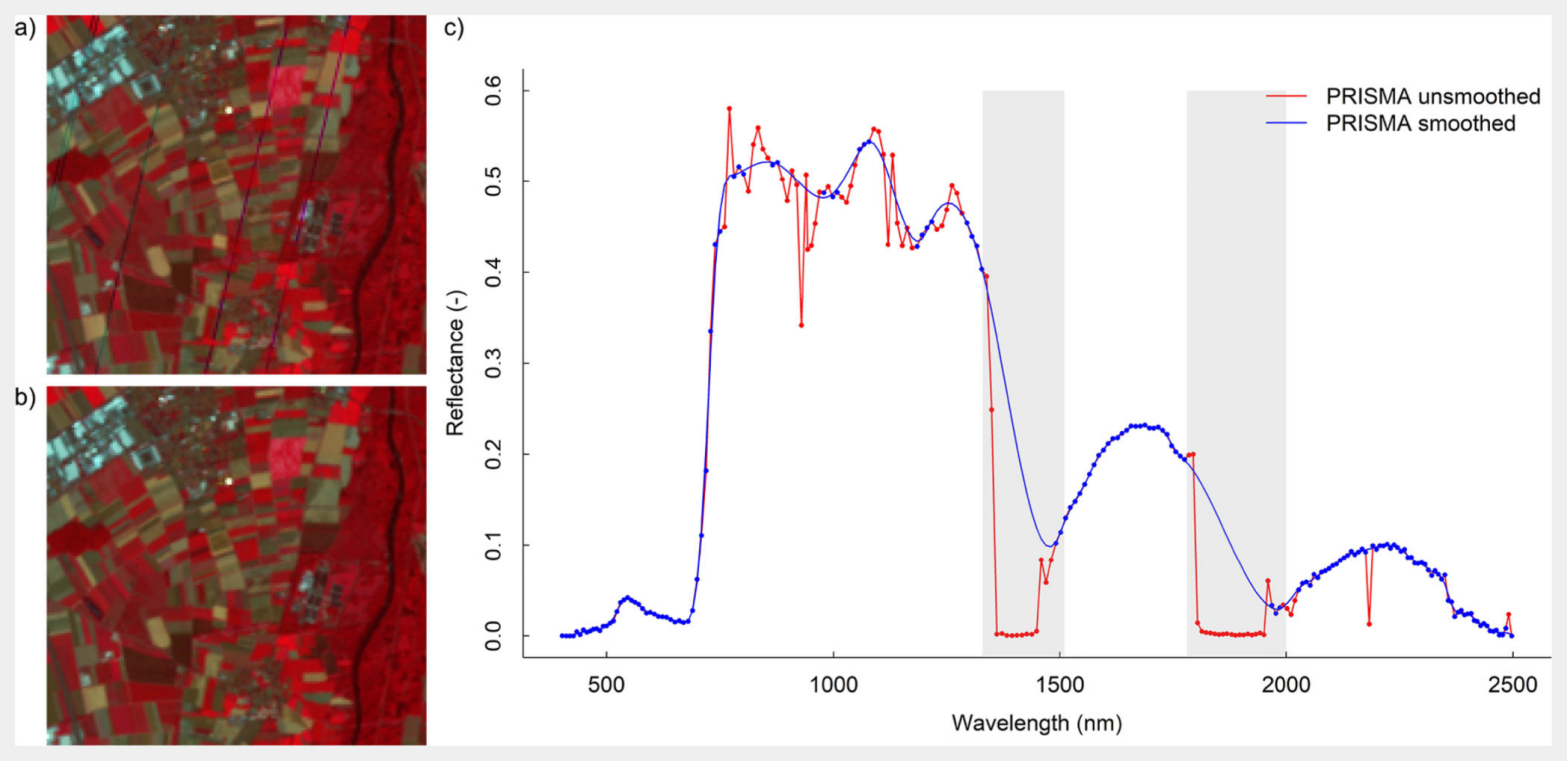
a) RGB false colour composition of a PRISMA subset before pre-processing, vertical stripes are clearly visible in the image; b) RGB false colour composition of a PRISMA subset after pre-processing; c) Example of a vegetation PRISMA spectra before (red line) and after (blue line) pre-processing. The red dots mark the bands that were removed by the smoothing procedure on this specific pixel. The blue dots indicate the bands used for the spline smoothing interpolation. The shaded grey areas indicate the spectral regions that were removed after the spline smoothing interpolation to obtain the final PRISMA spectra. (For interpretation of the references to colour in this figure legend, the reader is referred to the web version of this article.)

For mapping application, a PRISMA scene was acquired from the area in the North of Munich, Germany, on August 01, 2020 (see Fig. 1). Though the image could not be acquired simultaneously to the *in situ* data collection, it fully included the MNI test site and also other agricultural areas. With a GSD of 30 m, the spatial resolution of PRISMA corresponds to the future EnMAP and CHIME sensors. The standard L2D PRISMA reflectance image was pre-processed using different R packages (Team et al., 2013) to obtain smooth spectra. Firstly, the findpeaks function included in the pracma package (Borchers, 2015) was applied to each pixel to exclude random spikes occurring at specific wavelengths. The threshold for the peak detection was set to 0.018. Secondly, noisy spectral regions were systematically excluded (i.e., 535–550 nm, 755–780 nm, 810–855 nm, 885–970 nm, 1015–1050 nm, 1080–1165 nm, 1225–1285 nm, 1330–1490 nm, 1685–1700 nm, 1725–1750 nm, 1780–1960 nm, and 1990–2030 nm) based on the visual comparison against ground spectra collected on homogeneous targets (i.e., vegetation, asphalt, crop residues) with a field spectroradiometer (SR-4500; Spectral Evolution, USA). A spline smoothing interpolation was then applied using the SplineSmoothGapfilling function implemented in the FieldSpectroscopyCC package (Wutzler et al., 2016) to obtain cleaned PRISMA spectra. Finally, the atmospheric water absorption regions located at 1350–1510 nm and 1795–2000 nm were excluded. Fig. 2 illustrates the applied corrections on one examplarily PRISMA spectrum before and after cleaning and smoothing.

#### 2.1.3 Spectral equivalence of data sets

To ensure high equivalence of all spectral data sets (i.e., field spectrometer, PRISMA and simulated spectra, see also Section 2.2.1) some further processing steps were required. If in any dataset bands were of poor quality due to artifacts (e.g. noise in water absorption regions), these bands were subsequently removed from all (three) datasets. Altogether, some bands in the blue visible (400–470 nm), water absorption regions (1345–1510 and 1795–2000 nm) and at the upper limit of the SWIR range (2143–2500 nm) were excluded, leading to a total of 207 valid bands to be explored from 470 to 2143 nm. This slight reduction of the spectral range in particular in the SWIR is only a minor limitation, since most important spectral information is still available: in the study of Berger et al. (2020) a band selection algorithm was applied to investigate optimal spectral domains for CNC retrieval. The algorithm was based on the GPR property of automatic relevance determination (ARD) covariance using a wrapper strategy (Verrelst et al., 2016). All identified optimal spectral bands to estimate CNC from future EnMAP sensor data comprised those used in our study with one exception (2234 nm).

### 2.2 Theory, models and retrieval concept

**Fig. 3:**
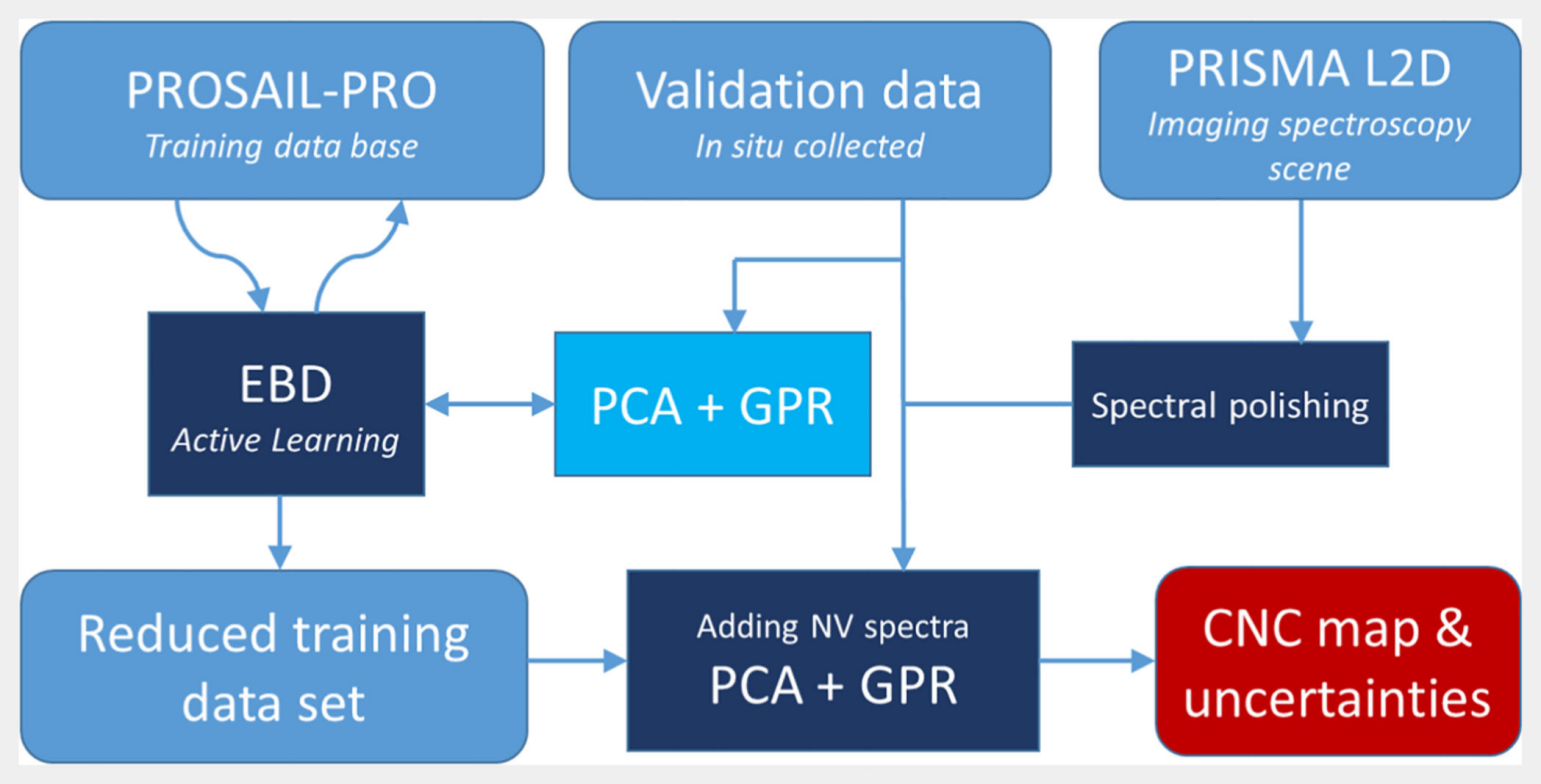
Workflow of hybrid mapping strategy for PRISMA CNC mapping. NV: non-vegetated.

The applied methodology is built upon foundations in leaf and canopy radiative transfer modeling in combination with concepts in the field of machine learning and imaging processing. The pursued work-flow is conceptualized in Fig. 3, and further elaborated in the sections below.

#### 2.2.1 Radiative transfer modeling

The essence of hybrid retrieval strategies for vegetation traits retrieval is that a ML regression algorithm is trained by simulated data coming from coupled leaf-canopy RTMs. In this way, a retrieval model is built for a specific variable from simulations covering a wide range of leaf-canopy states. The aim is to render the final model sufficiently generic for being applicable in an operational processing chain for global applications (Verrelst et al., 2015; Verrelst et al., 2019).

For the simulations, we used the PROSPECT-PRO leaf optical properties model (Féret et al., 2021) capable of separating leaf dry matter or leaf mass per unit leaf area (LMA) into leaf protein content (*C_p_*) and CBC. PROSPECT-PRO was coupled with the 1D canopy RTM Scattering by Arbitrarily Inclined Leaves, 4SAIL (Verhoef and Bach, 2007), to PROSAIL-PRO for generation of a training data base. The coupled model simulates reflectance at the canopy scale as a function of diverse biophysical (e.g. LAI and average leaf inclination angle) and leaf biochemical input parameters (e.g. *C_a_b, C_p_*, leaf carotenoid content or leaf equivalent water thickness). Briefly, 1000 combinations of PROSAIL-PRO model input parameters were randomly generated and corresponding reflectance was simulated. See Table 2 for sampling and ranges of model input parameters and the study of Berger et al. (2020) for full information about the generation of the training data base. Leaf nitrogen content can then be directly calculated from *C_p_* with the protein-to-nitrogen conversion factor of 4.43 (Yeoh and Wee, 1994), and LAI was used to upscale from leaf to canopy level (see Eq. 1). Finally, simulated “aboveground N content”, denoted here as CNC, in [g/m^2^], was added (Berger et al., 2020):

(1) $$CNC=\left(LAI\cdot C_{p}\cdot 10,000\right)/4.43$$

Bi-directional canopy reflectance was simulated with the PROSAIL-PRO model using PRISMA spectral configuration and excluding noisy bands as identified in the image pre-processing steps. In this way, simulated reflectance, *in situ* collected signatures and spaceborne imaging spectroscopy data were spectrally equivalent (see Section 2.1.3). Note that the size of the training dataset of 1000 samples (i.e. CNC with corresponding reflectance), which may appear small compared to classical look-up table approaches (e.g. Richter et al., 2009), is justified by the fact that a standard implementation of a GPR can not cope with thousands of samples within reasonable time. The processing time rises exponentially with increasing size as the computation involves the inversion of a N x N matrix, where N is the number of simulations (Rasmussen and Williams, 2006). Yet this apparent limitation is well compensated by the kernel-based algorithms, where for each estimation the new input is compared with all the (training) samples contained in the model. Hence, a GPR requires a relatively small training dataset to identify the nonlinear relationships between spectral observations and variables of interest, and then delivers highly competitive results compared to other machine learning methods, such as neural networks (Lazaro-Gredilla et al., 2014; Rivera-Caicedo et al., 2014). This has been confirmed by several studies using the same or similar sampling sizes for GPR training data sets, e.g. Upreti et al. (2019),Verrelst et al. (2020) and Pipia et al. (2021).

#### 2.2.2 Gaussian process regression

Gaussian process regression (Rasmussen and Williams, 2006) is chosen as core algorithm in the hybrid retrieval scheme since it has proven good performance in variable retrieval studies (Verrelst et al., 2012; Verrelst et al., 2013; Verrelst et al., 2015b). See also reviews of Verrelst et al. (2015, 2019, 2016) for a rationale of using GPR as opposed to alternative statistical methods.

Notationally, the GPR model establishes a relation between the input (*B*-bands spectra) **x** ∈ ℝ^*B*^ and the output variable (vegetation trait to be retrieved) *y* ∈ ℝ of the form (Equ. 2):

(2) $$\hat{y}=f(x)=\sum_{i=1}^{N}\alpha_{i}K\left(x_{i},x_{j}\right),$$

where ${\left\{x_{i}\right\}}_{i=1}^{N}$ are the spectra used in the training phase, *a_i_* ∈ ℝ is the weight assigned to each one of them, and *K* is a function evaluating the similarity between the test spectrum **x** and all *N* training spectra, $x_{i}={\left[x_{i}^{1},x_{i}^{2},\ldots ,x_{i}^{B}\right]}^{⊤}$, *i* = 1, ...,N. We used a scaled Gaussian kernel function (Equ. 3):

(3) $$K\left(x_{i},x_{j}\right)=\nu \exp\left(-\sum_{b=1}^{B}\frac{{\left(x_{i}^{b}-x_{j}^{b}\right)}^{2}}{2\sigma_{b}^{2}}\right)+\delta_{ij}\cdot \sigma_{n}^{2},$$

where *v* is a scaling factor, *B* is the number of bands, *σ_b_* is a dedicated parameter controlling the spread of the relations for each particular spectral band *b, σ_n_* is the noise standard deviation and *δ_ij_* is the Kro-necker’s symbol. The kernel is thus parametrized by signal *(v, σ_b_*) and noise (*σ_n_*) hyperparameters, collectively denoted as ***θ*** = {v, σ_b_, σ_n_}.

For training purposes, we assume that the observed variable is formed by noisy observations of the true underlying function *y* = *f* (**x**) + ∈. Moreover we assume the noise to be additive independently identically Gaussian distributed with zero mean and variance *σ_n_*. Let us define the stacked output values **y** = (*y*_x_*, y_n_*)^T^, the covariance terms of the test point k* = [k(x*,**x**j),...,k(x*,**x**_*n*_)]^T^, and k** = k(x*,x*) represents the self-similarity of x*. From the previous model assumption, the output values are distributed according to Equ. 4:

(4) $$\left(\begin{array}{c} y\\ f\left(x_{*}\right) \end{array}\right)\sim 𝒩\left(0,\left(\begin{array}{cc} K+\sigma_{n}^{2}I & k_{*}\\ k_{*}^{⊤} & k_{**} \end{array}\right)\right).$$

For prediction purposes, the GPR is obtained by computing the posterior distribution over the unknown output y*,p(y*|x*, 𝒟), where ***𝒟*** ≡{**x**_*n*_,*y_n_*| *n =* 1, ..., N} is the training dataset. Interestingly, this posterior can be shown to be a Gaussian distribution, $p\left(y_{*}|x_{*},𝒟\right)=𝒩\left(y_{*}|\mu_{{\text{GP}}_{*}},\sigma_{{\text{GP}}_{*}}^{2}\right)$, for which one can estimate the *predictive mean* (point-wise predictions), see Equ. 5:

(5) $$\mu_{\text{GP*}}=k_{⋆}^{⊤}{\left(K+\sigma_{n}^{2}I\right)}^{-1}y,$$

and the *predictive variance* (confidence intervals) as in Equ. 6:

(6) $$\sigma_{\text{GP*}}^{2}=k_{**}-k_{*}^{⊤}{\left(K+\sigma_{n}^{2}I\right)}^{-1}k_{*}.$$

The corresponding hyperparameters ***θ*** are typically selected by Type-II Maximum Likelihood, using the marginal likelihood (also called *evidence*) of the observations, which is also analytical. When the derivatives of the log-evidence are also analytical, which is often the case, conjugated gradient ascent is typically used for optimization (see Rasmussen and Williams (2006) for further details).

With respect to EO mapping applications, GPR is simple to train and works well with a relative small data set, as opposed to other methods like neural networks or random forests. GPR often outperformed these other non-parametric regression methods in remote sensing applications, which may be among others due to the use of the ARD kernel function rendering the model quite flexible. Furthermore, GPR provides information about the level of uncertainty (or confidence intervals) associated with the estimates, e.g. in form of a confidence map that provides insight in the robustness of the retrieval (Verrelst et al., 2013), and about the relevance of bands, which can be used for identifying the sensitive spectral regions (Verrelst et al., 2016; Camps-Valls et al., 2016; Camps-Valls et al., 2019). As a final remark, in this work we used the Matlab implementation of GPR as opposed to earlier works using codes coming directly from Rasmussen and Williams (2006), see also Verrelst et al. (2012,). The Matlab version offers a few extra options, such as a variety of kernel functions. These options enable to optimize the training phase more efficiently, leading to a gain in training run time (e.g., by using squared exponential kernel). While this gain is small when training a model only one time (in the order of seconds), it becomes substantial when GPR is implemented in an iterative process with AL.

#### 2.2.3 Active learning

AL aims to optimize training datasets through intelligent sampling by means of an iterative procedure. In the context of regression for terrestrial EO data analysis, AL techniques are typically categorized into two groups: *uncertainty* and *diversity* (Verrelst et al., 2016). In a recent survey (Berger et al., 2021) it was observed that choosing samples according to their diversity often led to optimal results. Particularly the Euclidean distance-based diversity (EBD) method was found top performing in most reviewed studies, and therefore we adapted this method for our study. The EBD method (Douak et al., 2013) selects those samples out of the pool that are distant from the already included ones in the training set, using squared Euclidean distance (Equ. 7):

(7) $$d_{E}={\left\|x_{u}-x_{l}\right\|}_{2}^{2},$$

where *x_u_* is a sample from the candidate set, and *x_l_* is a sample from the training set. All distances between samples are computed and then the most remote are selected. An additional optimization option was introduced in Verrelst et al. (2020). Thereby, the AL algorithm is run against external validation data. In this way, the training data base becomes optimized against real data. Both, *uncertainty* and *diversity* methods, have been implemented as an AL module into ARTMO’s machine learning regression algorithms toolbox (https://artmotoolbox.com/) (Rivera-Caicedo et al., 2014).

#### 2.2.4 Delineation of the hybrid retrieval workflow

Once having the training data and validation data prepared, the crucial part of developing generic and robust hybrid models can be found in the training strategy. Ultimately, the final model needs to comply with the following criteria, i.e. being: (i) able to deal with collinearity and noise present in hyperspectral data; (ii) able to provide fast and light models; (iii) generally applicable to a wide range of vegetation states for processing complete heterogeneous scenes. Accordingly, the following workflow was pursued: First, the spectral training data was compressed to 20 principal components using PCA, accounting for criterion (i) (Rivera-Caicedo et al., 2017). Based on earlier experiences with VSWIR hyperspectral data, 20 components were evaluated as an adequate trade-off between simplifying the training step while preserving the relevant spectral variability and thus information content of the variable of interest (De Grave et al., 2020; Danner et al., 2021). Second, in order to establish fast and light retrieval models (criterion ii), sampling reduction by AL methods was performed. A fast algorithm is essential when integrating the above step into the AL strategy, as it requires many iterations. Therefore, the training was done with the Matlab version of GPR representing the fastest option among all tested GPR versions. The efficient and fast EBD method was selected as most suitable AL method, see also Berger et al. (2021). Hence, from the pool of 1000 labeled samples (pairs of simulated reflectance and CNC), 10% were randomly selected as the initial training data set and the process was repeated for up to 1000 iterations. Following each iteration, a new sample was selected by the EBD method and added to the training data. When all distances between samples are computed, the sample with the largest distance is selected (Verrelst et al., 2016). In our procedure, this new sample was only added when performance improved as evaluated by the root mean square error (RMSE) against the provided validation data. The whole process was repeated until all samples of the training data set were evaluated. The corresponding goodness-of-fit statistics (e.g., RMSE, *R*^2^) for each sample subset were recorded.

The sampling strategy for generation of the training data set simulated by the PROSAIL-PRO model allows a wide range of vegetation states. However, an additional step was required to completely fulfill criterion (iii): To ensure that the model is able to deal with heterogeneous surfaces, 24 distinct non-vegetated spectra were added to the reduced training data set. These spectra were directly selected from the PRISMA image and covered all kinds of non-vegetated surfaces, including bare soil, water bodies and man-made surfaces. Given thatthese spectra are generally smoother and significantly different from the simulated vegetation spectra, the finally trained model should be able to sufficiently interpret the full spectral variability present in a hyper-spectral image, and thus to correctly infer the traits of interest. Additionally, it was tested whether adding Gaussian noise (ranging from 1 to 5%) to the spectra of the final training dataset would further optimize the performance, e.g., similar as in Brede et al. (2020), De Grave et al. (2020), Verrelst et al. (2020). Internal results suggested that noise did not improve retrieval performance, implying that the here developed AL-based models do not require artificial noise on simulated spectral datasets.

## 3 Results

### 3.1 Optimized sample selection for CNC modeling

**Fig. 4:**
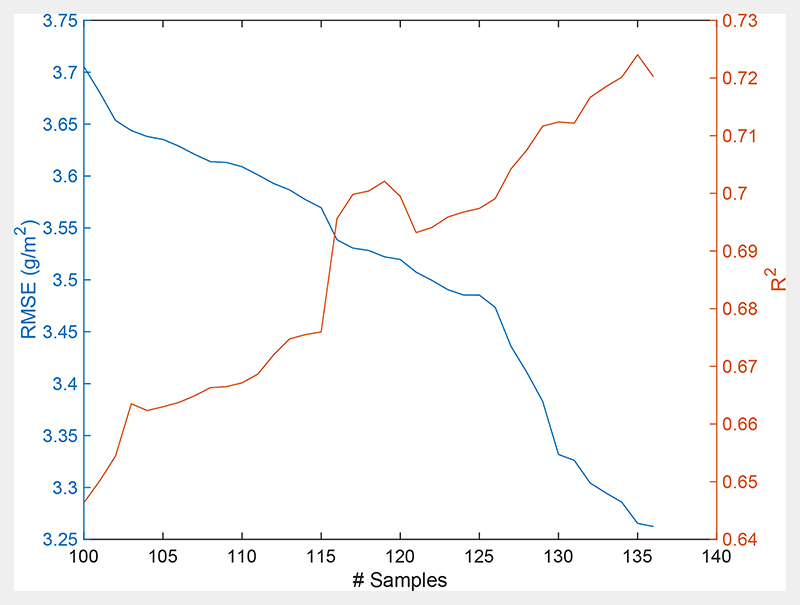
Goodness-of-fit results (RMSE, *R*^2^) using AL (EBD) against validation data. The AL sequence started with 100 samples and stopped with 136 samples.

A semi-supervised active learning strategy was applied to optimize the training samples for the GPR-based model building. Calculations relied on the EBD technique, with RMSE providing the selection criterion, as demonstrated in Fig. 4 (left Y-axis). Thereby, the EBD-GPR algorithm explored the full data pool, which was composed of 1000 simulations transformed to 20 PCA components with corresponding CNC values. The figure also nicely shows the effect of the training data size on final CNC models’ accuracy: starting with the initial dataset of 100 samples, each time one new sample was added by the algorithm, evaluated against *in situ* data and then only kept if the models’ accuracy improved. Whereas the sample-by-sample iteration process required a few minutes using the Matlab GPR, training the final model took merely a few seconds (see Table 3).

Optimal results were obtained after adding 36 samples to the 100 starting samples (RMSE: 3.26 g/m^2^; *R*^2^: 0.72). It should hereby be remarked that lowering the RMSE does not necessarily go along with an improvement of *R*^2^, as can be read on the right Y-axis of Fig. 4. Although it follows the same general trends as RMSE, the pattern provided by *R*^2^ is more irregular, indicating it as a less reliable measure than RMSE for AL testing. When repeating the procedure without AL (i.e., 1000 samples), it led to poorer validation results (RMSE: 6.40 g/m^2^; *R*^2^: 0.41) and a longer training time (appr. 18 s) (Table 3). Accordingly, the 136 samples selected by EBD were employed for training the CNC-GPR algorithm.

**Fig. 5:**
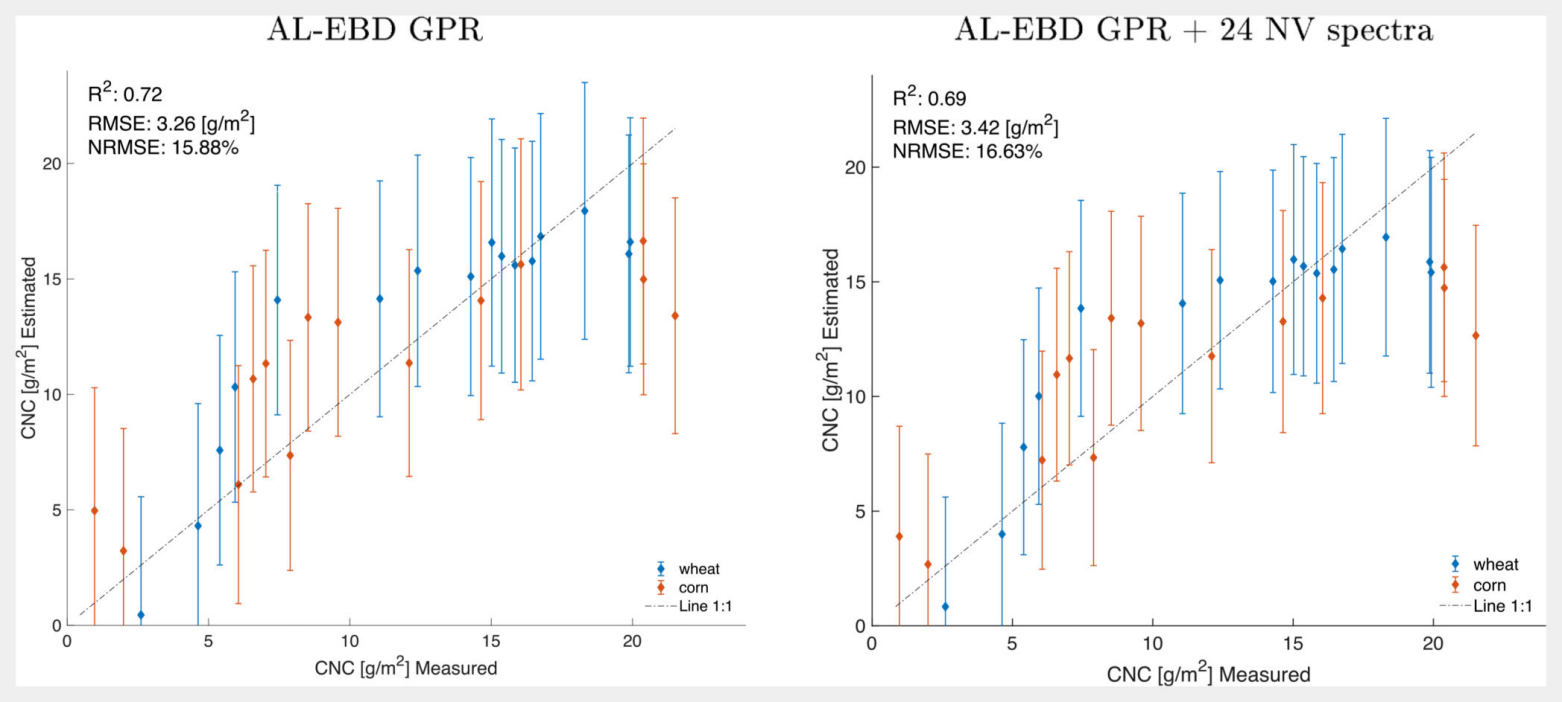
Measured vs estimated CNC along 1:1-line including uncertainty intervals for EBD-reduced training data set (left), and EBD-reduced + 24 added non-vegetated (NV) spectra (right).

Fig. 5 additionally compares the CNC model results when adding 24 non-vegetated spectra (Fig. 5, right), which led to slightly poorer validation results against *in situ* field data as opposed to without non-vegetated spectra (Fig. 5, left). Moreover, a stronger saturation effect occurs in case of the more heterogeneous training data set, mainly caused by the corn data at late mature growth stages. Nevertheless, this option was chosen for mapping the full heterogeneous PRISMA scene in order to fulfill criterion (iii), i.e. to ensure that the model is able to deal with a diversity of spectral signatures. Goodness-of-fit results of all three validation set ups are provided in Table 3.

**Fig. 6:**
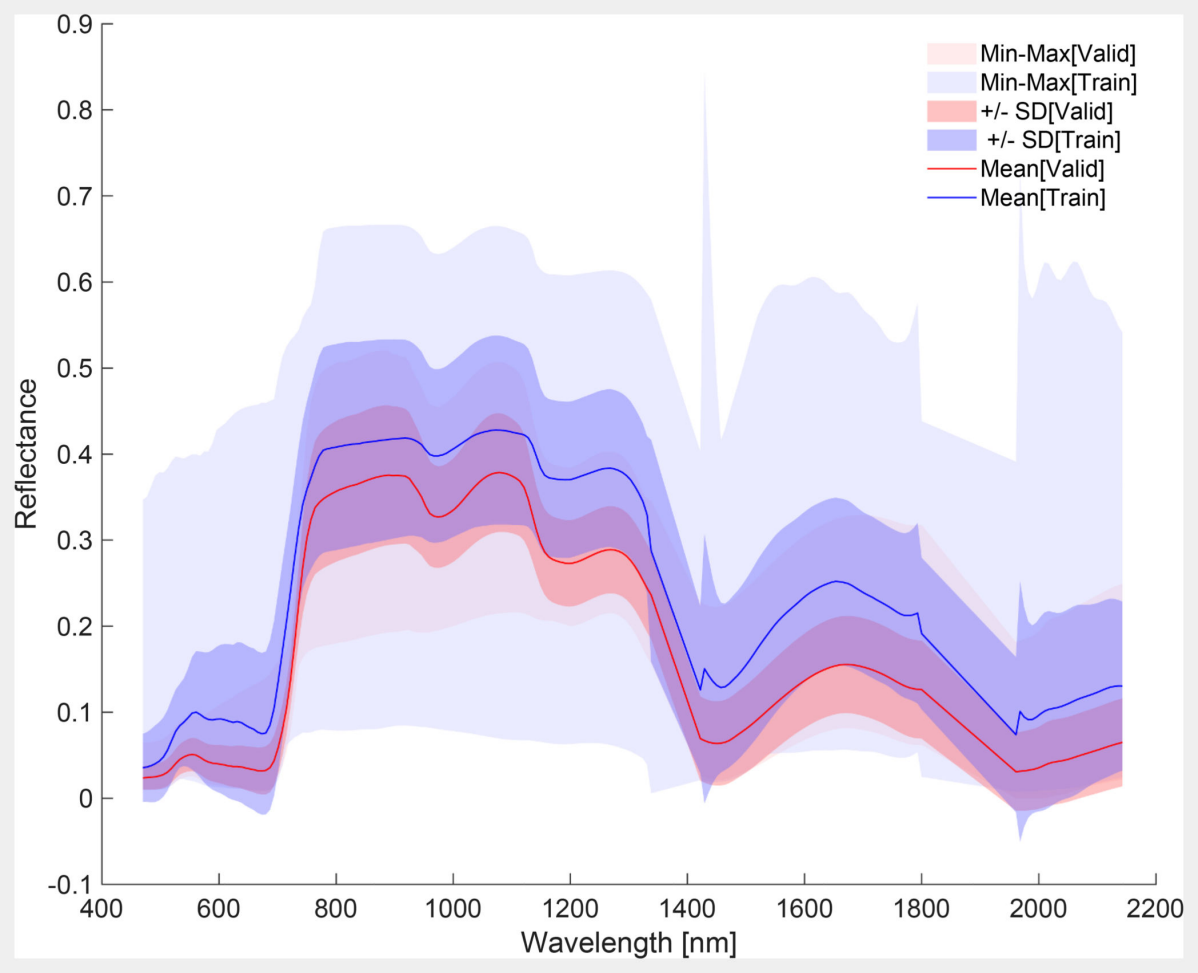
Statistics (mean, standard deviation, min–max) of EBD-reduced final training dataset (blue) vs. validation dataset (red). (For interpretation of the references to colour in this figure legend, the reader is referred to the web version of this article.)

In an attempt to inspect the spectral similarity between this final training data set (EBD with added non-vegetated spectra) and *in situ* validation dataset from MNI campaign, both data sets were statistically compared. Fig. 6 illustrates averaged spectra, with standard deviation and ranges in partly transparent colors. It can be observed that the training data match closely with the validation data. The broader range of the training data set implies a sufficient degree of generalization to ensure a generally-applicable model. Finally, the CNC-GPR model for mapping application is the result of applying both spectral dimensionality reduction (PCA with 20 components) and EBD-based sample reduction to the training data, with the addition of non-vegetated spectra (i.e., 160 samples in total) in order to accurately process scenes of heterogeneous landscapes.

### 3.2 Application of PRISMA imagery to CNC mapping

**Fig. 7:**
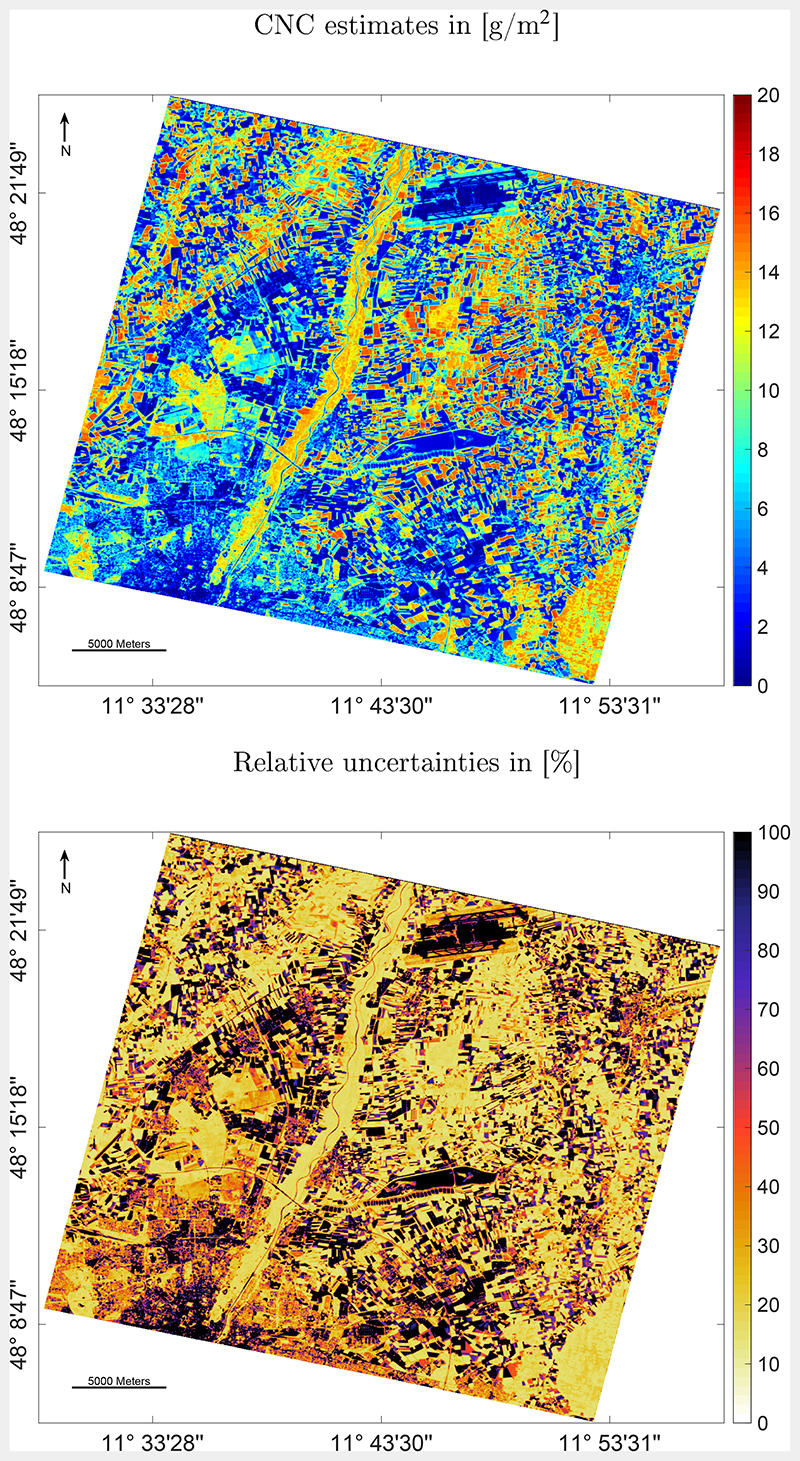
PRISMA image (01/08/2020) resampled to CHIME bands and processed into CNC in [g/m^2^] (top) with associated relative uncertainties in [%] (bottom).

Eventually, the final CNC-GPR model was applied to the PRISMA image over the area in the North of Munich. Since the model is so light, processing of the complete image (1237x1208 pixels) took merely four seconds as processed with a contemporary computer (Windows 10 Enterprise v.19041.572 64-bits OS, i7-9700 K CPI 3.60 GHz, 32 GB RAM). The scene covers a wide variety of different surface types, including the Northern part of the city of Munich, some lakes, the Munich airport, and the river Isar, flowing from South to North through the whole scene, with surrounding natural vegetation and forests (Fig. 7, top). The majority of the scene is characterized by areas of intense agricultural usage. The CNC map clearly shows the agricultural fields with high nitrogen content (Fig. 7, top). Highest values may be provided by corn fields, which reach a mature growth stage at the beginning of August. Also, the natural vegetation along the river and the forested area (in the bottom right) reveal rather high CNC. The map underneath (Fig. 7, bottom) demonstrates the associated relative uncertainties (expressed as coefficient of variation, in %). Generally, sufficiently low uncertainties are achieved, with higher values over the non-vegetated surfaces where CNC reaches close-to-zero values. Hence, the associated uncertainty map can serve as a quality layer, e.g. to exclude uncertain areas.

**Fig. 8:**
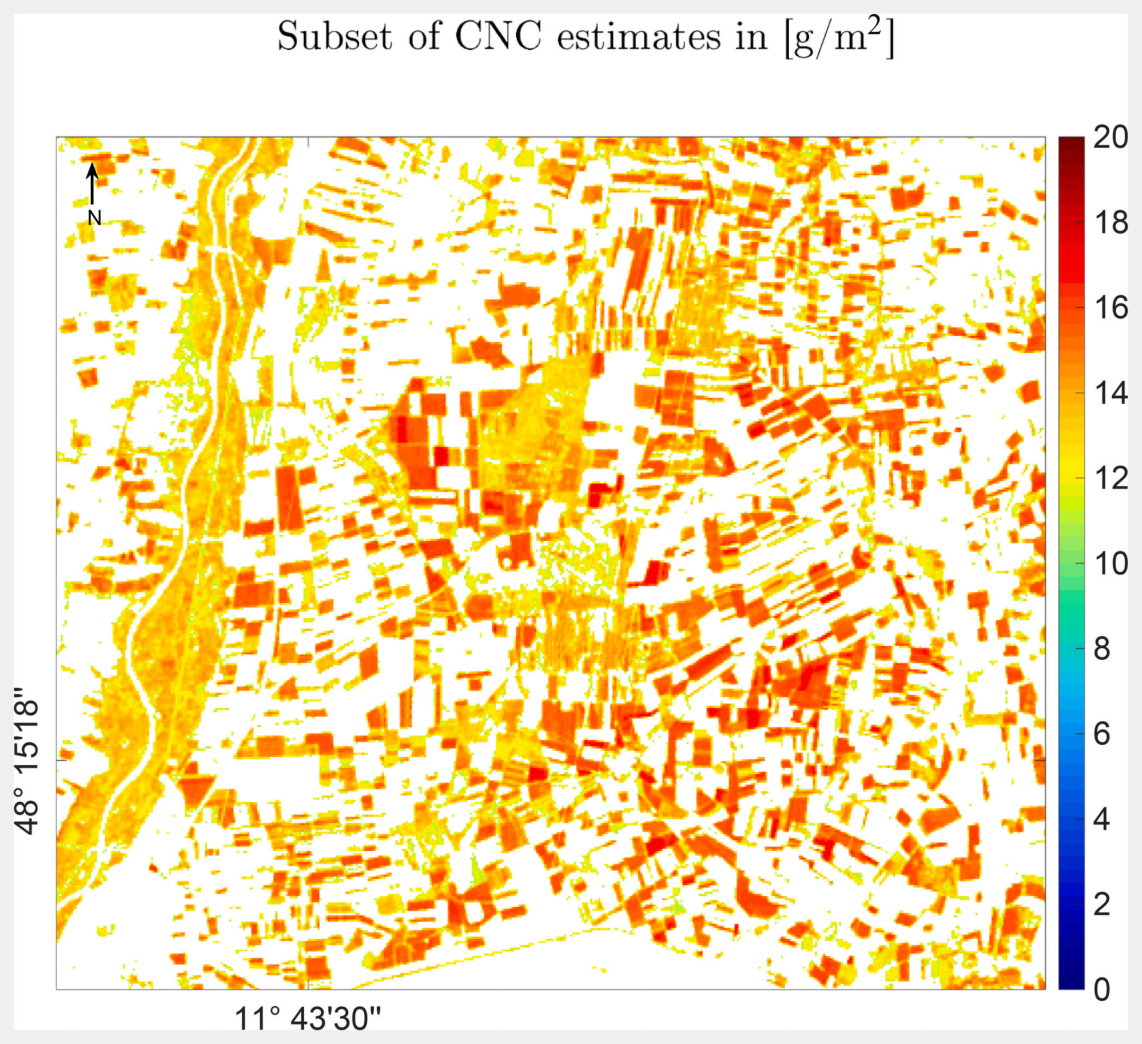
Subset of the PRISMA CNC map in [g/m^2^]. Areas with relative uncertainty of more than 20% were masked out.

Therefore, for a better interpretation of the CNC mapping result, a subset over such an agricultural area, including the MNI test site, was processed (Fig. 8), using the uncertainty information as a spatial mask. For this, a threshold of 20% as defined by the Global Climate Observing System (GCOS, 2011) was applied for exclusion of the most uncertain model results. With this threshold only the croplands with medium and high CNC are detected. Though no *in situ* CNC validation data is available yet, it is known that the corn field from 2018 (see Fig. 1) was again planted with corn in 2020. With CNC values around the maximum (20 g/m^2^), the map shows plausible estimates for the actual growing period. Further, the rather low uncertainty provided by the GPR retrieval model for the majority of this region provides fidelity in the obtained mapping results.

## 4 Discussion

### 4.1 Hybrid retrieval workflow

To enable CNC estimation from space, a hybrid retrieval workflow was proposed based on a simulated training data set, which was optimized by means of dimensionality reduction both in the sampling and spectral domains, and then trained with the probabilistic ML algorithm GPR. The applied workflow was based on an innovative leaf optical properties model (PROSPECT-PRO, Féret et al., 2021), using the spectral information of protein content to estimate CNC. This is in contrast to numerous previous attempts, which were based upon the relation of N and chlorophyll content, hence relying on spectral signals in the visible and near infrared domains (Hansen and Schjoerring, 2003; He et al., 2016; Tian et al., 2011). The feasibility of the protein-based approach using the SWIR spectral range has been demonstrated in two previous studies simulating EnMAP spectral configurations (Berger et al., 2020; Verrelst et al., 2020). As it was proposed in Verrelst et al. (2020), here we introduced the additional option of AL for efficient CNC estimation from a spaceborne hyperspectral acquisition.

A major challenge within any hybrid approach lies in the generation of training data for the ML regression algorithms. Although promising CNC retrieval results can be obtained with empirical data (Cilia et al., 2014; Pullanagari et al., 2016) or within an end-to-end processing chain using solely simulated data (Verrelst et al., 2021), applying a hybrid model on real, sometimes noisy data, is more challenging. Some studies suggested to add noise to the simulated (training) spectra, significantly improving prediction performance for biophysical variable retrievals (Danner et al., 2021; Brede et al., 2020). While adding noise can indeed account for different uncertainty sources, instead our results suggest that the implementation of intelligent sampling through AL methods can be key to tackle this problem, as it will be further discussed in the next section.

### 4.2 Mapping CNC from space

To assure high mapping accuracy, three different criteria have been applied to the proposed workflow, as also listed in Section 2.2.4. A first (i) requirement was to deal with collinearity and noise being typically present in hyperspectral data. To tackle these issues, PCA was chosen to reduce the dimensionality of the spectral dataset to a lower feature space, and to increase processing efficiency, ensuring at the same time a minimum of information loss (Jolliffe and Cadima, 2016). It seems advantageous to condense spectral information into components as opposed to relying on feature selection, as in Berger et al. (2020), yet this topic has to be further analyzed. The spectral information content used to establish components is surely higher than of single wavelengths. Moreover, the impact of noise can be better minimized when using feature transformation (or engineering), such as PCA, instead of single bands for model building. Several studies demonstrated that 20 components are more than sufficient to ensure high theoretical estimation accuracy for LAI when using GPR algorithms (Rivera-Caicedo et al., 2017; De Grave et al., 2020; Danner et al., 2021). In fact, the majority of variance (around 99%) of the training data is already covered within the first 10 components (e.g., in our final model over 99% was achieved with first seven components). At the same time, subtle but relevant information can be contained in the remaining components. If this is the case, then the ML algorithm (GPR) gives more relevance to it, though this comes with the risk that rather noisy information is exploited. Here, the CNC variable is composed of LAI and leaf protein content, with the latter having several subtle absorption features in the SWIR. Hence it was decided to keep this high number of components to ensure that maximized variance of the created features (components) is captured by the GPR model.

A second (ii) requirement is to provide fast and light models. While hybrid models are already fast as opposed to radiometric based model inversion strategies (e.g., see Verrelst et al., 2015b; Verrelst et al., 2015), further optimization can be achieved with AL. Earlier studies with AL techniques in hybrid workflows demonstrated that with intelligent sampling the training data set could be adapted to the variable of interest, which led to smaller training data sets, and thus lighter and faster retrieval models (Verrelst et al., 2016; Verrelst et al., 2020; Upreti et al., 2019; Pipia et al., 2021; Berger et al., 2021), which also facilitates storage within software toolboxes. As further benefit, the AL algorithm EBD proved to be a successful strategy to optimize the training dataset without the need to add artificial noise to the spectral data. In our study, a range of Gaussian noise did not lead to further improvements, which is remarkable, as in prior studies this was always an essential step to adapt hybrid models to cope with (noisy) real space-based reflectance data (e. g. De Grave et al., 2020; Estévez et al., 2020). Moreover, GPR trained over AL-reduced datasets provided not only higher retrieval results but also lower uncertainties as opposed to training with full data pools (Berger et al., 2021). This was also confirmed by our results when using the full training data set without AL: the resulting CNC map showed relative uncertainties over 100% for the majority of pixels (not shown). Besides, image processing was more than three times slower (appr. 13 s). See also Berger et al. (2021) for a more in-depth discussion on the advantages of using AL heuristics in hybrid retrieval models.

A third (iii) requirement is to strive for developing models that are generally applicable to a wide range of vegetation states, hence being robust and generic for mapping full heterogeneous landscapes. Among others, this can be achieved with AL. To avoid overspecialization, a 10% initial dataset was applied within the AL procedure, which is a higher share compared to the initial data used by prior AL studies (Verrelst et al., 2016; Verrelst et al., 2020). Internal tests suggested that this initial 10% to start AL is an optimal trade-off to balance the final model between being sufficiently generic and adequately adapted to real data (results not shown). Since the AL sampling selection is run against *in situ* data, it is also essential that the field dataset is sufficiently broad. Hence, the collection of good quality field data remains an important part of the retrieval algorithm development. Fig. 6 indicates that the CNC model was trained over a wide range of vegetation states well embracing the *in situ* data set. To cope with spectral heterogeneity within a scene, the CNC model was trained with additional non-vegetated spectra (coming from bare soil, water, man-made surfaces, etc.), which led to slightly poorer validation results against *in situ* data. This is not surprising given that these added spectra broadened the training data set to unknown spectra compared to the *in situ* data base. Yet, it is an essential part of developing a generally applicable hybrid model for achieving meaningful image-wide retrievals (see also examples with Sentinel-2 (Estévez et al., 2020; Estévez et al., 2021) and −3 (De Grave et al., 2020)).

Optimization of the above-mentioned criteria led to the final CNC-GPR model that was applied to the PRISMA image. The obtained CNC map clearly shows the agricultural fields with high CNC, fields and natural vegetation with medium CNC, and low to non-vegetated surfaces with hardly any CNC. Although there was no *in situ* ground data set for validation available at the time of the image acquisition, plausibility of a mapping result can be also interpreted without physical validation. For instance, the relatively narrow intra-field distributions of the estimated variable along with spatially consistent mapping results are an indirect measure for the accuracy of the model, see also discussion in Atzberger and Richter (2012). Moreover, the obtained map can be interpreted together with the associated uncertainty provided by the GPR (Fig. 7, bottom). This map provides confidence in the CNC map, with overall low relative uncertainties, i.e. below 20%. Uncertainty information provided by GPR models is an attractive feature, as it can be used to mask out uncertain areas and to provide corresponding maps (see Fig. 8). Additionally, uncertainty maps can be used to identify spectra of surface types not considered in the training database (Verrelst et al., 2013). For instance, uncertainties could assist in deciding whether complementary training data is required, e.g. to account for very complex vegetation structures. Likewise, it could be identified whether spectral signatures from non-vegetated surfaces should be added to improve the training data set and enhance confidence of the mapping accuracy. Furthermore, the associated uncertainties provide information of the models’ transferability when applying the model to images at different locations and from other observation dates. The possibility to quantify the transferability is the very essence for selecting GPR as the core retrieval algorithm of hybrid model developments. When uncertainties stay below a certain threshold, the requirement for time-consuming ground campaigns for collection of *in situ* reference data for model evaluation can be minimized (Verrelst et al., 2013).

Previous attempts to estimate CNC from spaceborne imaging spectroscopy, in particular from Hyperion sensor data, led to similar or less good results (Abdel-Rahman et al., 2013; Miphokasap and Wannasiri, 2018; Tian et al., 2011). For instance, Abdel-Rahman et al. (2013) tested random forest (RF) regression algorithms for prediction of sugarcane leaf N concentration, achieving R^2^ of 0.67. In the study by Miphokasap and Wannasiri (2018), stepwise multiple linear regression (SMLR) and support vector regression (SVR) were applied to establish CNC retrieval models, leading to R^2^ from 0.67 to 0.78. Although these studies already demonstrated feasibility of CNC estimation from spaceborne imaging spectroscopy data, the applied models were calibrated exclusively on *in situ* collected N data, meaning that these models were limited to specific sensor data, vegetation type and geographical location. Moreover, no full map over the whole landscape was provided. Further, the experimental character of these studies strongly limits the comparability of the results with the here presented generic model. Mainly, they relied on the apparent correlation between chlorophyll content and N (Tian et al., 2011). This assumption has certain operational advantages, e.g. in respect of the strong spectral chlorophyll absorption features in the visible wavelength range, which is typically covered by optical sensors. Nevertheless, quantitative non-destructive retrieval of N content via the leaf proteins is expected to be more reliable and robust due to the strong linkage between plant protein and nitrogen contents (Berger et al., 2020; Féret et al., 2021). This linkage is mainly to be found in the SWIR region due to several absorption features corresponding to proteins (i.e. between 1500 nm and 2400 nm), and in the NIR domain with two additional features (910 nm and 1020 nm) (Curran, 1989; Berger et al., 2020; Féret et al., 2021).

### 4.3 Study limitations and future challenges

Despite being developed in a hybrid framework based on a broad range of simulated data, a main limitation of the current CNC model version is that validation and AL tuning was only possible against one *in situ* dataset. Ideally, reference data should be acquired over multiple vegetation types and integrated into the GPR-AL workflow in order to provide more robust and generic models. Nonetheless, such data are rarely available. The intense campaign carried out at MNI site during two growing seasons and on two crop types was extremely labourintensive and the organ-specific N samples can be considered as trustful and high quality measurements. Further, it must be noted that the relatively high retrieval accuracy could only be obtained against measured CNC of crop leaves plus stalks. With hyperspectral data it is hard to detect the N content of fruits, in particular of thick corn cobs, since solar radiation can not penetrate thick tissues and thus is not able to transport information about the N (or other biochemicals) contained within corn cobs or wheat ears (Wocher et al., 2018). This was demonstrated on the same data base in the study by Berger et al. (2020), where the inclusion of fruit N content led to underestimation by the developed CNC models. Note that in contrast to most previous studies, we concentrated on the estimation of the area-based N content (in g/m^2^) instead of the mass-based measure (i.e., N%). N content is a very important trait from a physiological perspective relating N to photosynthesis and carbon acquisition (Evans, 1989). Moreover, it allows to upscale leaf N to the canopy level and is not influenced by the dilution phenomenon as N%. For these reasons, it was recommended to retrieve the area-based measure to describe optimal N status (Berger et al., 2020; Baret et al., 2007).

Further efforts are foreseen to maximize the performances of developed CNC models. For instance, optimal spectral dimensionality of the training dataset should be examined. In this respect, it is planned to apply PCA to specific spectral regions and test out the information content of individual components. In this context, also the retrieval accuracy as function of the number of components will be tested against larger CNC field data sets. Moreover, due to the relatively large range of possible sun zenith angles at different geographic latitudes, it would be worth to explore whether information of SZA should be included as additional training feature for the ML algorithms, e.g. similar as the neural network models implemented in the biophysical processor toolbox of the Sentinel Application Platform (SNAP, Weiss and Baret, 2016).

Finally, near-term satellite imaging spectroscopy missions will advance our understanding of physiological processes and stimulate further progress in functional vegetation traits retrieval and mapping applications. In this context, instead of relying on proximal sensing spectroscopy data, future AL (EBD) methods should be run against spaceborne imaging spectroscopy data associated with field measurements for model tuning. For instance, from 2022 onwards, the EnMAP satellite (Guanter et al., 2015) is expected to co-exist with PRISMA, which will strongly enhance the availability of hyperspectral time series data along with other complementary missions. Therefore, it would also be important to harmonize the spatial resolutions of the spaceborne sensors with field measurements and model simulations. Given these improvements pending, consolidating spatially-harmonized and spectrally-equivalent time series products can open a promising path for progressing towards routine delivery of next-generation global CNC products, e.g. as delivered through the future CHIME mission.

### 4.4 Perspectives for CHIME

Lastly, the proposed workflow for CNC mapping is currently under investigation within the framework of the planned operational CHIME mission. Foreseen to become part of the Copernicus fleet, CHIME shall provide free access to routinely acquired Level 1B, 1C and 2A products. Along with other vegetation traits models, the CNC model has been implemented into CHIME’s end-to-end (E2E) mission performance simulator (Verrelst et al., 2021). E2E instrument simulators are software tools developed to support satellite mission preparatory activities (Kerekes and Landgrebe, 1989; Kerekes and Baum, 2002; Segl et al., 2012; Vicent et al., 2016). CHIME’s E2E simulator is able to simulate realistically and very accurately the complete chain starting from data recording, sensor calibration and data pre-processing to sensor products up to final surface properties maps, including vegetation traits. In the E2E simulator, multiple scenarios can be introduced and validated, e.g. varying topography and heterogeneous surfaces, extreme weather and atmospheric events, varying sensor or instrumental configurations. One of the main advantages of the E2E simulator is that any of the generated products can be validated per-pixel against reference input maps. With the concurrent availability of real imaging spectroscopy data, real and simulated validation exercises will allow adjustments and improvements of the developed vegetation traits models. Hence, the here presented CNC model is not necessarily the final model to be integrated into the CHIME processing chain, and further improvements are already in progress, such as the exploitation of the full CHIME spectral range and validation and tuning against new data from planned campaigns. Over the course of the upcoming years, improved versions are expected to be developed, until the mission is launched, and likely beyond. Eventually, the E2E processing chain intends to serve as a processor benchmark for implementation into an operational processing chain. As such, once the processing chain is in place, and the mission launched, the CNC model, along with other vegetation traits models (Verrelst et al., 2021; Berger et al., 2021), will enable a quasi-instantaneous extraction of a range of vegetation products together with associated uncertainties from L2 reflectance data.

## 5 Conclusions

In this study we developed a novel workflow for operational mapping of canopy nitrogen content designed for spaceborne imaging spectroscopy missions. The workflow builds upon a hybrid method that combines advanced RTM and machine learning approaches to ensure sufficient general applicability and fast processing. The hybrid method is based on simulations coming from PROSAIL-PRO used for training of a GPR algorithm. The usage of GPR provides the additional advantage of delivering associated uncertainties together with the CNC estimates. To customize the CNC-GPR model towards handling successfully real PRISMA data, it was required to: (1) make use of dimensionality reduction method PCA to condense the spectral data into components, (2) explore an active learning technique to specialize and optimize the training data set, and (3) add non-vegetated spectra to the final training data base to provide generic models for mapping heterogeneous landscapes. The CNC-GPR model was validated with a high accuracy (RMSE of 3.4 g/m^2^) and was subsequently applied to a PRISMA image over the North of Munich. Overall, relatively low uncertainty of the estimates was obtained, suggesting transferability of the retrieval model to other space and times. With the presented workflow the first CNC map was produced from spaceborne imaging spectroscopy data, providing a path towards routinely monitoring of canopy nitrogen content over agricultural areas in a globally-applicable operational framework.

## Figures and Tables

**Table 1:** Statistics of *in situ* samples: crop type, dates, number of measurements (No.), range of BBCH growth stages, measured ranges, mean and SD (in brackets) of leaves plus stalks N content of winter wheat *(Triticum aestivum*) and corn (*Zea mays*) at MNI location.

Crop/Date	No.	BBCH	Range in [g/m^2^]	N content in [g/m^2^]
*Triticum aestivum*, 29/03–06/07 2017	9	25–83	2.6–19.9	13.9 (5.8)
*Triticum aestivum*, 12/04–13/07 2018	7	28–87	4.6–16.5	10.9 (4.6)
*Zea mays*, 13/06–15/09 2017	8	13–85	0.9–12.1	6.6 (3.5)
*Zea mays*, 15/06–22/08 2018	6	30–87	7.9–21.5	16.8 (4.7)

**Table 2:** Parameterization of leaf (PROSPECT-PRO) and canopy (4SAIL) models, with notations, units, ranges and distributions of inputs used to simulate the spectral training database. $\bar{x}$: mean, SD: standard deviation. Ranges and distributions come from Berger et al. (2020).

Model variables		Units	Range (min-max)	Distribution
*Leaf variables (PROSPECT – PRO):*				
*N*	Leaf structure parameter	unitless	1.0–2.5	Uniform
*C_ab_*	Leaf chlorophyll content	[*μg/* cm^2^]	0–80	Uniform
*C_w_*	Leaf water content	[cm]	0.001–0.02	Uniform
*C_xc_*	Leaf carotenoid content	[*μ*g/cm^2^]	0–15	Uniform
*C_anth_*	Leaf anthocyanin content	[*μ*g/cm^2^]	0–2	Uniform
*C_p_*	Leaf protein content	[g/cm^2^]	0.001–0.0025	Gaussian ($\bar{x}$:0.0015, SD: 0.0005)
CBC	Carbon-based constituents	[g/cm^2^]	0.001–0.01	Uniform
*Canopy variables* (4SAIL):				
LAI	Leaf area index	[m^2^/m^2^]	0.1–7	Gaussian ($\bar{x}$: 3, SD: 2)
*α_soil_*	Soil scaling factor	unitless	0–1	Uniform
ALA	Average leaf angle	[°]	30–70	Uniform
HotS	Hot spot parameter	[m/m]	0.01–0.5	Uniform
SZA	Sun zenith angle	[°]	30	
OZA	Observer zenith angle	[°]	0	

**Table 3:** Goodness-of-fit statistics of CNC-GPR models as trained with EBD-reduced training data set with and without 24 non-vegetated (NV) spectra, as well as training with full data pool, all validated against MNI *in situ* data. CPU time for training and testing (seconds).

Model set up	RMSE (g/m^2^)	NRMSE (%)	R^2^	train (s)	test (s)
GPR	3.26	15.88	0.72	0.57	0.0061
GPR + 24NV spectra	3.42	16.63	0.69	2.18	0.0044
GPR full	6.40	31.49	0.41	17.93	0.0038
